# Sudden death from long-standing benign ventricular parasystole after atrial fibrillation ablation

**DOI:** 10.1016/j.hrcr.2025.06.004

**Published:** 2025-06-04

**Authors:** Yui Koyanagi, Yuya Nakamura, Takamasa Ishikawa, Shuhei Arai, Taku Asano, Toshiro Shinke

**Affiliations:** Division of Cardiology, Department of Medicine, Showa University School of Medicine, Tokyo, Japan

**Keywords:** Ventricular parasystole, Premature ventricular contractions, Torsades de pointes, Ventricular fibrillation, QT prolongation, Atrial fibrillation ablation, Sudden cardiac death

**Key Teaching Points**

• Parasystolic premature ventricular contractions (PVCs), although typically regarded as benign, may become malignant triggers when transient proarrhythmic factors such as electrolyte disturbances are present.
• Dynamic changes in rhythm or autonomic tone, such as those occurring after atrial fibrillation ablation, may transiently increase vulnerability to arrhythmias initiated by parasystolic PVCs.
• Recognition of parasystolic patterns, especially when accompanied by modifiable risk factors, should prompt heightened vigilance, aggressive electrolyte correction, and proactive monitoring.

## Introduction

Premature ventricular contractions (PVCs) are often considered benign, particularly in structurally normal hearts. However, under specific electrophysiological conditions, such as variable coupling intervals, electrolyte abnormalities, and QT prolongation, even seemingly benign ectopy can become malignant. Among these, parasystolic PVCs originating from a protected ectopic focus and discharging independently of sinus rhythm, may serve as potent triggers of torsades de pointes (TdP) or ventricular fibrillation (VF).^1^ Recent literature has emphasized the association between ventricular parasystole and sudden cardiac death, even in the absence of overt structural heart disease.^2^

We report a rare and fatal case of VF triggered by an R-on-T PVC with parasystolic characteristics in a woman who had recently undergone pulmonary vein isolation using pulsed field ablation (PFA). This case underscores the arrhythmogenic potential of parasystolic PVCs, the difficulty in identifying their malignant nature, and the opportunities for timely intervention.

## Case presentation

**Figure 1:**
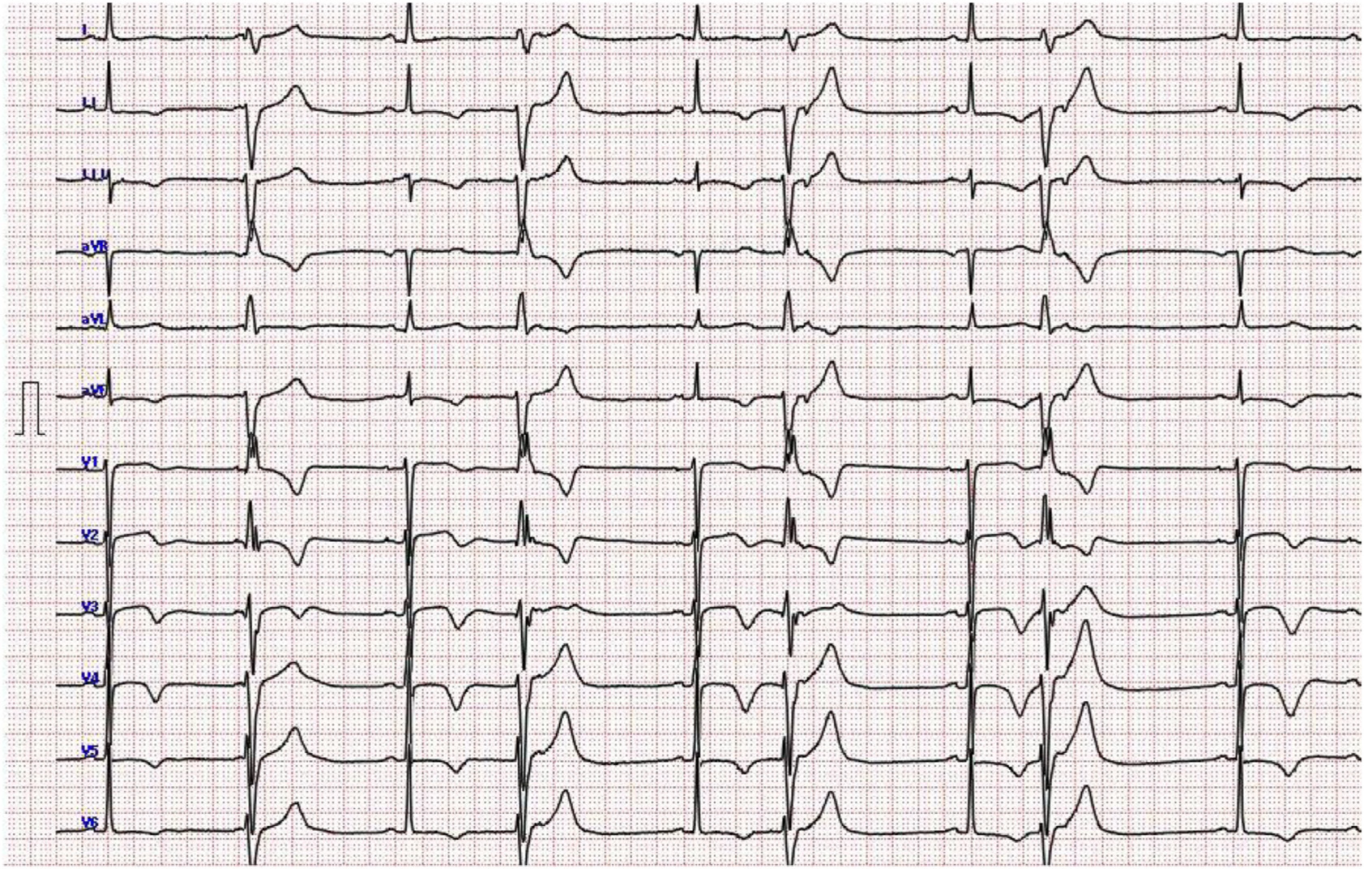
Frequent parasystolic premature ventricular contractions (PVCs) with right bundle branch block morphology and a superior axis. The PVCs exhibit fixed interectopic intervals and variable coupling intervals relative to preceding sinus beats, fulfilling the criteria for classic ventricular parasystole.

**Figure 2:**
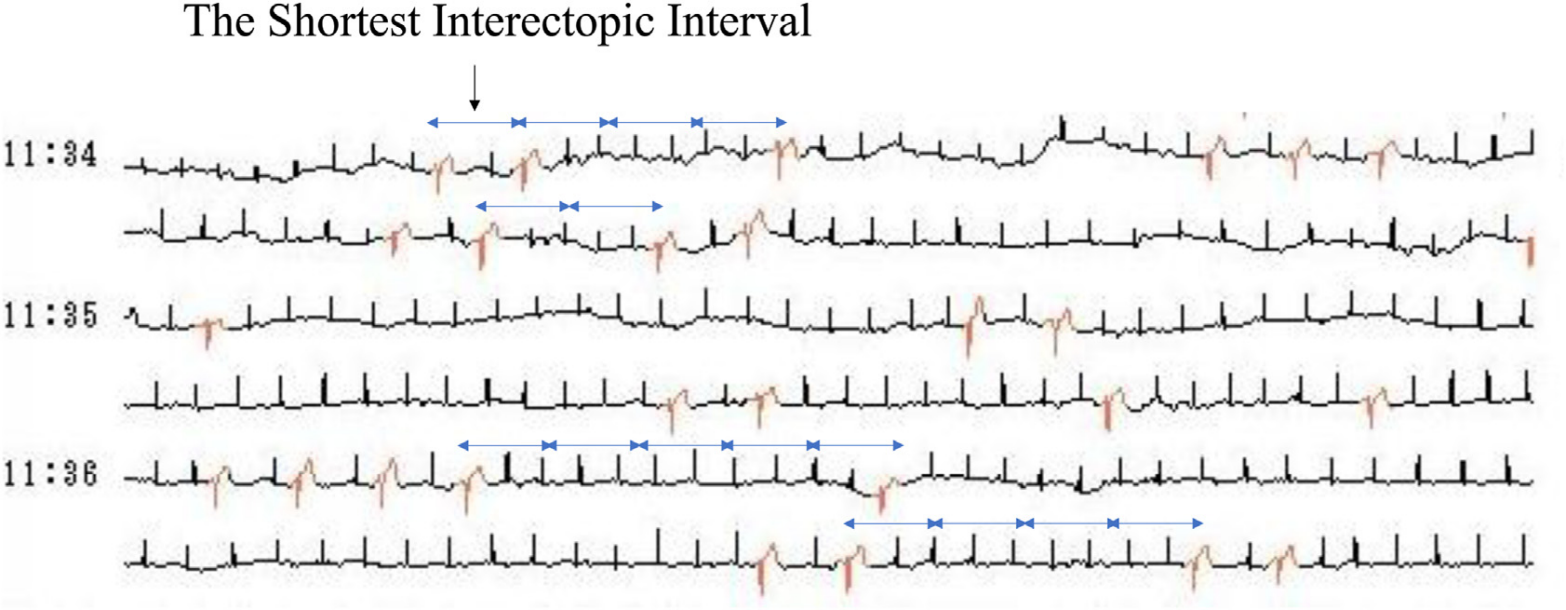
Continuous Holter electrocardiogram (ECG) monitoring demonstrating frequent premature ventricular contractions (PVCs) with fixed interectopic intervals. These PVCs occurred as integer multiples of the shortest interectopic interval, fulfilling a diagnostic criterion for classic ventricular parasystole.

A 68-year-old woman with no history of structural heart disease had been noted to have frequent PVCs. A 12-lead electrocardiogram (ECG) showed PVCs with right bundle branch block morphology and a superior axis (Figure 1). Twenty-four–hour Holter monitoring performed 12 years earlier showed that PVCs accounted for 11% of total beats. On the basis of these findings, ectopy was suspected to represent ventricular parasystole (Figure 2). However, as the patient remained asymptomatic, she was managed with watchful observation without specific treatment.

**Figure 3:**
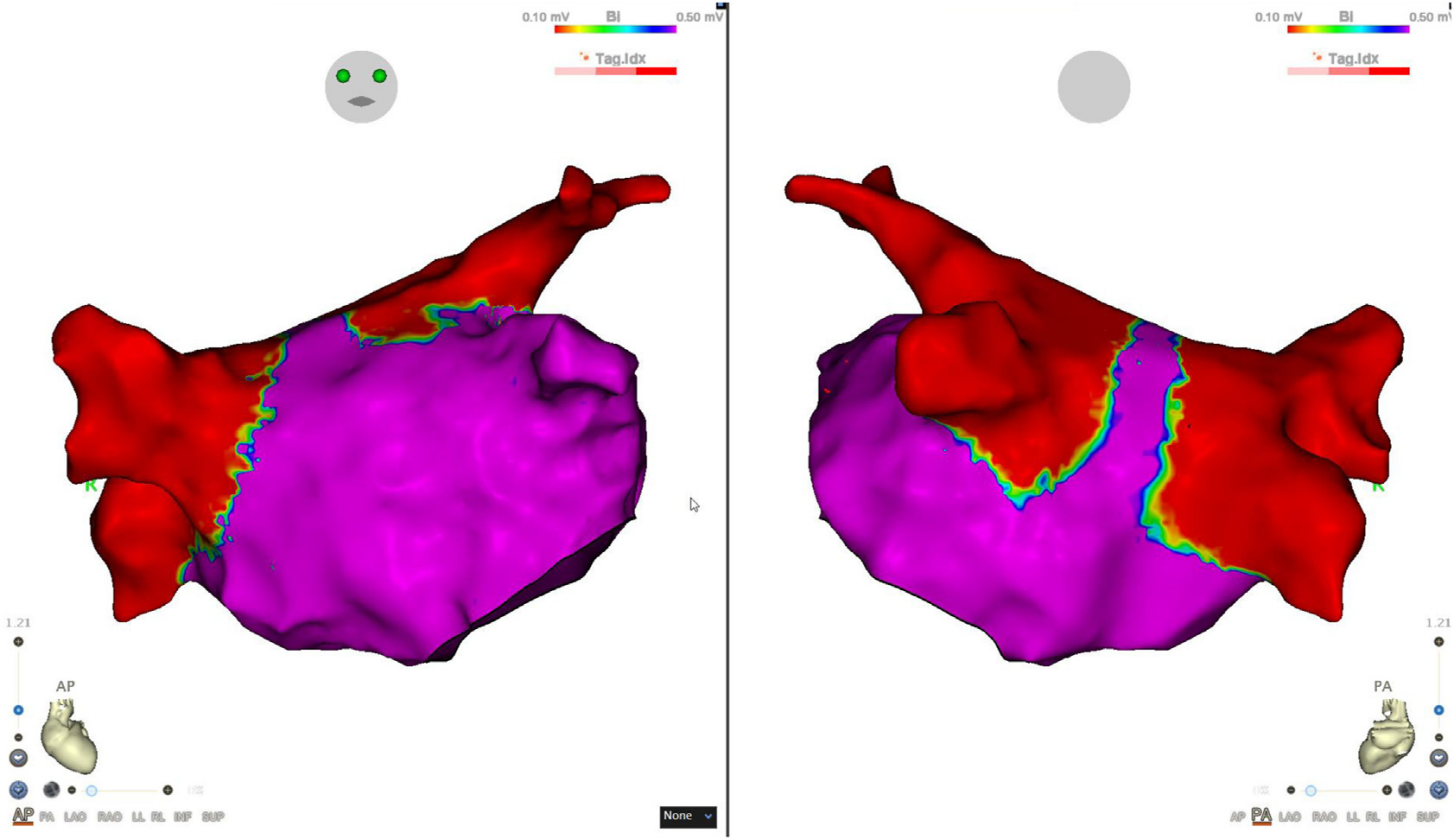
Postablation electroanatomic map showing successful bilateral pulmonary vein isolation. Electroanatomic mapping performed after catheter ablation demonstrates complete isolation of all 4 pulmonary veins. AP = anteroposterior; PA = posteroanterior.

She was referred to our institution with symptomatic persistent atrial fibrillation and was scheduled for catheter ablation. She underwent bilateral pulmonary vein isolation using PFA. Each of the 4 pulmonary veins received 8 applications (a total of 32 applications). The postablation electroanatomic map confirmed successful isolation (Figure 3). The procedure was completed without complication. Postprocedure transthoracic echocardiography showed no pericardial effusion and a preserved left ventricular ejection fraction of 62%. Laboratory testing revealed no signs of hemolysis. Her serum potassium level at that time was 3.4 mEq/L. The patient was not taking any antiarrhythmic drugs known to prolong the QT interval, and she remained in sinus rhythm after the procedure and was discharged on postoperative day 3 in stable condition.

**Figure 4:**
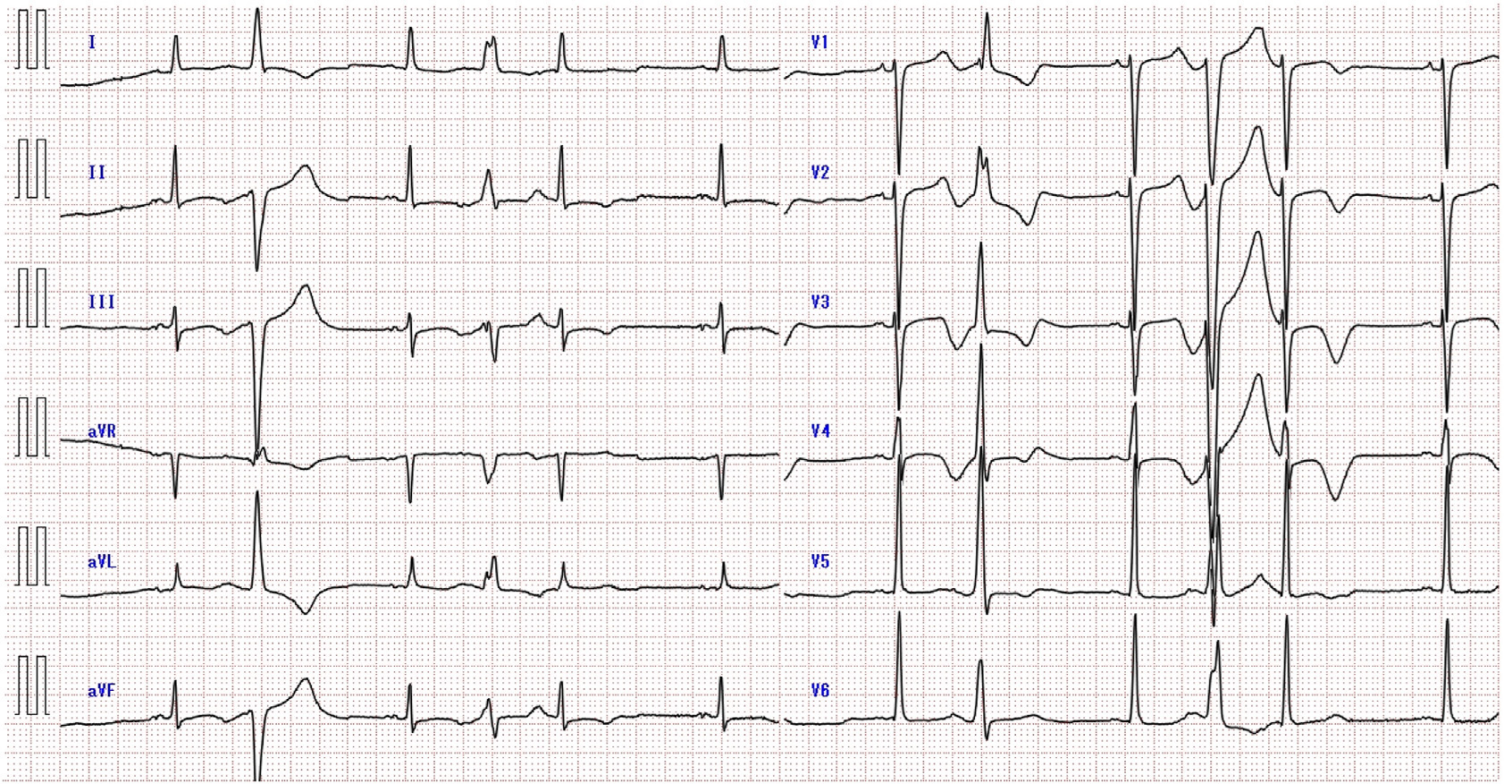
Twelve-lead electrocardiogram (ECG) recorded in the emergency department 2 days after discharge, demonstrating frequent monomorphic premature ventricular contractions (PVCs) with mild QT prolongation. The QT interval was 510 ms, and the corrected QT interval (calculated using Bazett’s formula) was 480 ms at a heart rate of 53 beats/min. This ECG was obtained during an episode of viral gastroenteritis with hypokalemia.

Two days after discharge, the patient presented to the emergency department with multiple episodes of diarrhea and palpitations. She was diagnosed with viral gastroenteritis. Laboratory testing revealed a mild elevation in inflammatory markers and a low serum potassium level of 3.3 mEq/L. A 12-lead ECG recorded at that visit showed frequent monomorphic PVCs, with a QT interval of 510 ms and a corrected QT interval of 480 ms (calculated using Bazett’s formula) at a heart rate of 53 beats/min (Figure 4).

**Figure 5:**
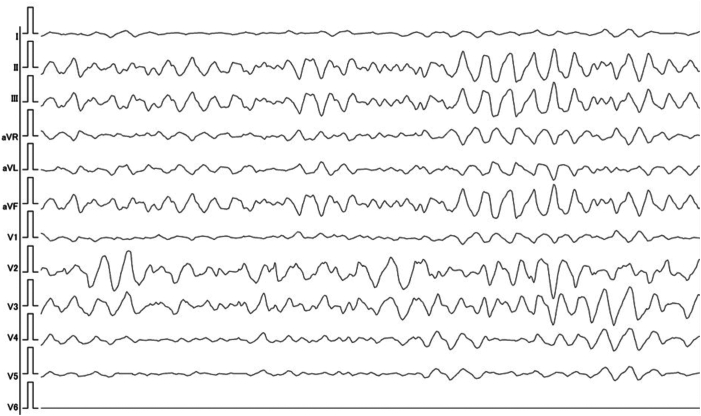
Twelve-lead electrocardiogram (ECG) recorded in the catheterization laboratory immediately after venoarterial extracorporeal membrane oxygenation initiation, showing ventricular fibrillation (VF). The patient was comatose and experiencing recurrent VF at the time of recording, requiring ongoing resuscitation efforts.

On the sixth day after discharge, she developed palpitations followed by a sudden collapse at home. Emergency medical services found her in VF, confirmed by a prehospital rhythm monitor. She received 2 external defibrillations during transport and was brought to our emergency department. Upon arrival, she was comatose and experienced recurrent VF (Figure 5). Emergent venoarterial extracorporeal membrane oxygenation was initiated. Emergent coronary angiography revealed no obstructive coronary lesions. Venoarterial extracorporeal membrane oxygenation support became unsustainable because of chest trauma and hemorrhage related to cardiopulmonary resuscitation efforts, resulting in death on hospital day 2. Autopsy, including gross and microscopic examination, revealed no evidence of structural heart disease or myocarditis.

## Discussion

Parasystole is traditionally considered a benign electrophysiological phenomenon, characterized by PVCs arising from a protected ectopic focus that discharges at a constant intrinsic rate, independent of the sinus rhythm. Surface ECG criteria for classic ventricular parasystole include at least 3 PVCs of identical morphology, fixed interectopic intervals, and variable coupling intervals relative to preceding sinus beats. In our case, the PVCs preceding VF fulfilled all 3 classical criteria, providing strong support for the diagnosis.

Retrospective review also revealed that the ECG obtained during her emergency department visit for diarrhea and palpitations captured the onset of a short-long-short sequence, initiated by a parasystolic PVC (Figure 4). It is conceivable that the palpitations she experienced just prior to collapse were early manifestations of TdP, which subsequently degenerated into VF. This case represents a rare but instructive example of sudden cardiac death triggered by parasystolic PVCs in a patient without structural heart disease. Detailed analysis of the prearrest rhythm strip demonstrated that the initial short cycle of the short-long-short sequence was caused by a parasystolic PVC. This parasystolic PVC, followed by a compensatory pause (long cycle), and another short cycle with a PVC falling on the T wave—an R-on-T phenomenon—immediately initiated polymorphic ventricular tachycardia consistent with TdP, which rapidly degenerated into VF. Of note, as shown in Figure 1, the PVCs preceding VF exhibited variable coupling intervals ranging from 582 to 1110 ms. This resulted in a coupling interval variability of 528 ms, which far exceeds the 60-ms threshold associated with increased arrhythmic risk, as previously reported.^3^ Furthermore, these PVCs demonstrated a fixed interectopic interval, consistent with the definition of classic ventricular parasystole.

Importantly, the patient had remained completely asymptomatic despite frequent parasystolic PVCs over the previous 12 years, highlighting that even a mildly prolonged corrected QT interval (480 ms) in combination with ectopy can serve as a potent trigger for TdP under specific electrophysiological conditions, such as electrolyte disturbances and recent rhythm changes. Recent studies have challenged the traditionally benign perception of ventricular parasystole. Notably, Do and colleagues^2^ demonstrated that parasystolic PVCs were significantly associated with VF and progressive conduction system abnormalities in patients with cardiomyopathy. These findings suggest that parasystolic ectopy may serve as a clinical surrogate marker for occult His-Purkinje system injury, even in structurally normal hearts. In our patient, although autopsy did not reveal any gross structural abnormalities, the arrhythmic event was considered to have occurred in the setting of unstable electrophysiological conditions, including hypokalemia, hypomagnesemia, and recent rhythm changes. The short-long-short sequence immediately preceding VF onset in our patient warrants particular emphasis. The short-long-short sequence is known to promote early afterdepolarizations by prolonging repolarization, thereby increasing susceptibility to triggered activity and malignant ventricular arrhythmias.^4^ In this case, parasystolic PVCs—appearing with variable coupling intervals—dynamically interacted with underlying repolarization instability to generate a short-long-short pattern, culminating in TdP initiation via an R-on-T mechanism. This interplay highlights that parasystolic ectopy, even when relatively infrequent, can act as a lethal arrhythmic trigger under specific electrophysiological conditions.

From a clinical standpoint, this case emphasizes the importance of heightened vigilance when parasystolic PVCs are identified, particularly in the presence of transient proarrhythmic factors such as electrolyte disturbances or recent rhythm conversion. Although the patient had documented frequent PVCs before and after atrial fibrillation ablation, their malignant potential was not initially suspected. In retrospect, hospitalization during the episode of diarrhea-associated hypokalemia and hypomagnesemia, aggressive correction of electrolyte abnormalities, closer ECG monitoring, and early initiation of pharmacological therapies aimed at suppressing ectopy could have been considered to mitigate arrhythmic risk.

In summary, this case illustrates that parasystolic PVCs, traditionally regarded as benign, may in fact represent arrhythmogenic triggers capable of initiating fatal ventricular arrhythmias.^5^ Clinicians should recognize parasystolic patterns as potential markers of increased arrhythmic risk, particularly in dynamic clinical settings where repolarization instability is enhanced. Prompt identification, correction of modifiable risk factors, and vigilant surveillance may provide opportunities to prevent catastrophic outcomes.

### Limitations and clinical implications

This report has several limitations. Although the diagnosis of parasystole was supported by surface ECG criteria, electrophysiology study was not performed. The patient developed diarrhea on the fourth day after PFA ablation, despite no history of gastrointestinal disease. Although vagal plexus injury has been implicated in postprocedural diarrhea after radiofrequency ablation,^6^^,^^7^ no such association has been reported with PFA. Further studies are needed to clarify whether a similar mechanism may be involved. It must also be acknowledged that recognizing parasystolic PVCs as malignant triggers in a structurally normal heart is inherently difficult. Without clear R-on-T coupling or documented TdP, such PVCs are often dismissed as benign. This case underscores the importance of heightened vigilance when parasystolic patterns are identified, especially in the setting of electrolyte disturbances, autonomic fluctuations, or recent rhythm conversion.

## Conclusion

This case illustrates the potentially fatal consequences of parasystolic PVCs under vulnerable repolarization conditions. Even in the absence of structural heart disease, parasystolic PVCs with variable coupling intervals and short-long-short sequences should be regarded as potentially high risk. Early recognition of these arrhythmogenic patterns, correction of modifiable risk factors, and appropriate monitoring may offer opportunities for prevention. This case highlights the need for clinicians to reassess the malignant potential of so-called “benign” ectopy, particularly in the setting of transient electrophysiological instability.

## References

[1] Robles de Medina E.O., Delmar M., Sicouri S., Jalife J. (1989). Modulated parasystole as a mechanism of ventricular ectopic activity leading to ventricular fibrillation. Am J Cardiol.

[2] Do D.H., O’Meara K., Lee J. (2023). Ventricular parasystole in cardiomyopathy patients: a link between His-Purkinje system damage and ventricular fibrillation. JACC Clin Electrophysiol.

[3] Bradfield J.S., Homsi M., Shivkumar K., Miller J.M. (2014). Coupling interval variability differentiates ventricular ectopic complexes arising in the aortic sinus of Valsalva and great cardiac vein from other sources: mechanistic and arrhythmic risk implications. J Am Coll Cardiol.

[4] Viskin S., Schwartz A.L., Levi Y., Hochstadt A., Rosso R. (2020). Ventricular fibrillation after ablation of a benign arrhythmia: angry Purkinje syndrome?. HeartRhythm Case Rep.

[5] Steinfurt J., Asbach S., Odening K.E. (2018). Fascicular parasystole and recurrent syncope—a case report. Eur Heart J Case Rep.

[6] Jacobs V., May H.T., Crandall B.G. (2018). Vagus nerve injury symptoms after catheter ablation for atrial fibrillation. Pacing Clin Electrophysiol.

[7] Yamane T., Inaba O., Hachisuka E., Yamashita S., Yoshimura M., Nitta J. (2021). Persistent diarrhea following catheter ablation for atrial fibrillation: a lesser-known complication of left atrial ablation procedures. HeartRhythm Case Rep.

